# Promoting Cultural Humility by Integrating Health Equity Literature into the Pharmacy Curriculum

**DOI:** 10.3390/pharmacy10050116

**Published:** 2022-09-21

**Authors:** Vincent J. Venditto, Kristie Colón

**Affiliations:** College of Pharmacy, University of Kentucky, Lexington, KY 40536, USA

**Keywords:** cultural humility, health equity, journal club, social determinants of health, unconscious bias

## Abstract

Strategies that introduce students to unconscious bias and social determinants of health (SDOH) are critical to develop them as effective health care providers. We developed a semester-long activity that utilizes disease-relevant scientific literature to implement cultural humility training in a second-year rheumatology pharmacy course. Students were first re-introduced to implicit bias and then completed an anonymous survey at the beginning and conclusion of the course using a 5-point Likert scale to assess their perceptions of the role of biases and SDOH in patient care. Throughout the semester, five journal articles were assigned that relate to course material and focus on one characteristic (e.g., gout—gender). Students’ evolved perceptions of SDOH were compared to baseline data and characteristics of assigned articles indicate an improved understanding of SDOH including race/ethnicity (3.0 to 4.4, *p* < 0.0001); gender (2.8 to 4.0, *p* < 0.0001); and religion (2.3 to 2.9, *p* < 0.01). Among characteristics that were not directly discussed in the assignments, only education showed a significant increase (3.0 to 3.6, *p* < 0.01). Scientific articles that focus on health inequities relevant to course-specific diseases provide a strategy to integrate discussions that help students evaluate their biases and SDOH with the goal of improving patient care.

## 1. Introduction

Cultural humility is an essential aspect of any community that hopes to shift one’s perspective to focus on others’ experiences and needs. The shift to an empathic mindset is a critical and ethical component of healthcare, empowering clinicians to understand and respectfully communicate with their team members and patients to provide fair and equitable care [1]. Cultural humility training in healthcare colleges provides students with the necessary resources to ensure that race, ethnicity, religion, ability, gender, and other identities are accounted for when engaging with patients and considering a treatment plan. Furthermore, complications arise in patient care when unconscious biases persist, as well as limited life experiences or lack of awareness of how social determinants of health influence disease and subsequent treatment [2].

Clinical examples of social determinants of health in patient care continue to be examined and have been reported in one example in which gender and racial biases in pain assessment and treatment exist [3,4,5]. For example, such biases lead to worse post-operative outcomes for female patients treated by male physicians [5]. These findings are unacceptable for any clinician and necessitate the need to address these shortcomings for the duration of one’s educational experience, focusing on promoting a lifelong commitment of self-evaluation to engage with all communities effectively [6]. The necessity for lifelong learning in this area calls into question the term cultural competence, which suggests that it has an achievable endpoint, like memorizing medication dosing guidelines. Thus, cultural humility is a more appropriate term that offers more than merely being aware of cultural differences. Cultural humility is a lifelong learning framework requiring constant self-assessment and critique to identify and address systematic inequities [6]. As educators, we cannot expect our students to understand cultural humility without directly addressing healthcare inequity and examining how structural inequity disproportionately segregates marginalized groups from equal access and opportunity to care. Given the importance of cultural humility training in healthcare colleges, many institutions are starting to implement training to enhance students’ perceptions of bias [2,7,8,9,10].

Lifelong learning is not a new concept in the pharmacy curricula, as most coursework is designed to provide students with a framework for continued learning throughout their careers [11,12]. Although this is often facilitated with continuing education activities, engagement with the scientific literature is also a critical component of the pharmacy profession to apply current research into practice [13,14,15]. By bridging evidence-based practice strategies with cultural humility training, students are able to learn the necessary skills to read and engage with scientific literature that complements the disease-specific course materials. Herein, we describe the implementation of such exercises throughout a Pharmacy course in 2020 and 2021 and the quantitative data revealing how students’ perceptions of social determinants of health (SDOH) are impacted by the scientific literature that the students engage with.

## 2. Materials and Methods

### 2.1. Overview

Two consecutive cohorts of second-year pharmacy students enrolled in the Integrated Drugs and Diseases (IDD): Rheumatology course (*n* = 270) were assigned anonymous surveys and articles with an associated cultural humility aspect relevant to the course materials. Details of the surveys and reading assignments are provided in the subsequent subsections. This set of assignments was linked to a discussion of unconscious bias and pain management during the second week of class in which students first completed the age Harvard Implicit Association Test (IAT) at home to re-introduce the concept of bias, which was first formally presented to the students during orientation. The Harvard IAT was originally designed to measure the strength of associations between characteristics and stereotypes and serves as a mechanism to initiate discussions about bias. In class during the second week, students completed a baseline survey of their perceptions on how specific patient characteristics affect rheumatology care, followed by an in-class discussion of bias and SDOH. During subsequent weeks, students were assigned five articles to read. Students were asked to complete a survey after reading each paper to assess their perceptions of the articles. At the conclusion of the eight-week course, students were once again asked to complete the same survey administered during the second week of the course to assess their changing perceptions of SDOH. A comparison of the baseline data from the second week and their final perceptions of SDOH was immediately displayed for the students to observe how their perceptions had or had not changed. The UK Institutional Review Board has approved this study as exempt (#75846).

### 2.2. Baseline and Concluding Survey

During the second and final weeks of the course, students were given a survey on Mentimeter (Mentimeter AB, Stockhom, Sweden) which asked: How much would bias in the following categories affect rheumatology treatment? Using a 5-point Likert scale (1 = Strongly disagree, 5 = Strongly agree), students provided their perceptions of the following biases: Ability, Age, Economic Status, Education, Gender, Occupation, Race/Ethnicity, Religion. The bias characteristics used here were selected based on the Harvard IAT offerings.

### 2.3. Reading Assignments

Five assignments were selected for students to read and engage with using Perusall (Perusall, Austin, TX, USA), which is a social learning platform that drive collaboration around course content. Perusall is a social learning software that enables students to digitally annotate readings and interact with peers and course instructors, while also providing a platform for assigning points based on the level of student engagement. Articles were selected based on data collected from the survey administered during the second week of year 1 and assigned during both years. Articles were selected in disease states relevant to course materials to address one of the bias characteristics from the initial survey. Importantly, given that this would be one of the first times that some students would be engaging with scientific literature, articles selected for these assignments were somewhat easy to comprehend, and the selection is based on article length and data presented. The selected articles focus on the health disparities related to rheumatoid arthritis (RA) and socioeconomic status [16], osteoarthritis (OA) and religion [17], gout and gender differences [18], lupus and race [19], and and psoriatic arthritis (PsA) and skin color [20]. For clarity, the gout reference refers to sex differences based on HMO data [18], however, gender is a more appropriate term in this case, and we refer to this as gender differences. Perusall was utilized to assess active reading time and engagement with the articles and their peers. Students achieved full scores by meeting the threshold for active reading as well as a specific number of comments and responses. Reading assignments were due prior to the class period in which they would be discussed as a group. Discussions included an overview of the data presented and a highlight of the SDOH discussed within each paper. During this time students were provided opportunities to discuss what they viewed as the strengths and weaknesses of each manuscript and limitations to the studies, while also commenting on whether their perceptions had changed by reading each article.

### 2.4. Post-Assignment Surveys

In conjunction with the reading assignments, students were required to complete a survey of the assignment to assess their satisfaction with the article and their perceptions of the biases discussed in each paper. During the first year of this activity, the survey was conducted as an anonymous Mentimeter survey in class and not linked to their grade. Mentimeter is an audience engagement software for anonymous participation. During the second year of the activity, surveys were linked to course grades and completed using quizzes within the Canvas learning management system. The change in grading was implemented due to poor survey responses during year one. Questions in this survey included: (1) Did you like this paper?; (2) Why did you or did you not like this paper?; and (3) How much do you agree or disagree that the following characteristics played a role in this paper? Questions 1 and 2 provided insight into the utility of the activity, whereas Question 3 was related to the question on the baseline survey using a 5-point Likert scale (1 = Strongly disagree, 5 = Strongly Agree) to assess students’ perceptions of Ability, Age, Economic Status, Education, Gender, Occupation, Race/Ethnicity, and Religion as a SDOH.

### 2.5. Statistical Analysis

Data were analyzed using GraphPad Prism (GraphPad Software, San Diego, CA, USA), and statistical analyses were conducted using a Kolmogorov–Smirnov student’s *t*-test to assess the primary objective of longitudinal changes within the cohort after completing each of the assignments. Given that the data were collected in two separate cohorts, a cohort-to-cohort comparison was performed to also assess differences in baseline perception data. Statistical significance is defined here as *p* < 0.05.

## 3. Results

### 3.1. Students Have Different Baseline Perceptions of How Certain Characteristics Affect Rheumatology Care

To assess the students’ baseline perceptions of how different characteristics affect patient care in rheumatology, an anonymous survey was conducted in Mentimeter in which 96 out of 136 students (70%) and 112 out of 134 students (84%) responded during consecutive years. The survey data are summarized in Table 1 and ranked from highest to lowest based on year two. A statistically significant difference exists for the student’s perceptions of how ability, economic status, and religion affect patient care in rheumatology, suggesting that not all student cohorts enter with the same perceptions. Although such differences are evident between cohorts, age, ability, economic status, and occupation are perceived to be the strongest characteristics impacting patient care in both cohorts. Notably, both cohorts of students completed the age Harvard IAT prior to completing this survey, which likely primed their response to suggest that age is the strongest characteristic influencing rheumatology care.

### 3.2. Students Enjoyed Engaging with the Articles and Thought Critically about the Content

Based on the data from year one in which race/ethnicity, gender, and religion were lower scoring categories, scientific literature was identified for students to read to help them question their own perceptions. In particular, papers related to race/ethnicity were selected for lupus and PsA modules [19,20], gender was selected for the gout module [18], and religion for the OA module [17]. Given the similar trend in year two, the same papers were assigned in both years [16,17,18,19,20]. Students completed surveys to assess their relative enjoyment, while also identifying what characteristics were discussed in each paper. Although assessing enjoyment of the papers was not an objective of this activity, it was an important component of continuing to implement this activity in future years. In year one, a low response rate for surveys associated with each paper precluded appropriate analysis and comparison between cohorts. In year two, surveys were linked to grades and response rates to each paper-specific survey were near 100%, which enabled further analysis of student perceptions. In general, students in year two indicated that they enjoyed the papers that were assigned with 80–89% of students responding positively. Although anonymous survey response rates in year one was low throughout the semester (46–78%), students offered lower positive reviews of the articles (58–81%). The low response rates preclude proper comparisons within or between cohorts, but the lower positive reviews for papers in year one may be attributed to the anonymous survey, which enabled students to speak more freely about the assignments. Alternatively, frustration from the COVID-19 pandemic, or Zoom/Perusall/Mentimeter fatigue may have also led to the observed results.

During year two, students who liked the articles often indicated that they learned something or were intrigued by the data and sought additional literature to read more on the topic. Those who did not like the papers often pointed out a shortcoming of the paper, which led to their overall skepticism of the content. Though the papers rated well with students during year two, the gout paper, which touches on gender differences in gout care, had the lowest rate of students who indicated that they enjoyed the paper (80%). Of the students who indicated that they did not like the paper, 7 out of 29 students (24%) indicated that they were not interested in the topic, 5 out of 29 students (17%) commented on the writing or clarity of the message, and 59% indicated that there was a technical issue with the data that caused them to question the content. Despite the fact that the student commentary of the technical shortcomings of each study were valid, many comments related to the technical limitations of the data sets or patient populations investigated. Nevertheless, these data indicated that students were properly engaging with the articles and being critical evaluators of the scientific literature. Though this was not the primary goal of this activity, secondary benefits are possible, and there are opportunities to formally examine how this assignment encourages students to think critically and critique literature appropriately.

### 3.3. Student Perceptions of the Impact of Social Determinants of Health Evolved with Assigned Readings

**Figure 1:**
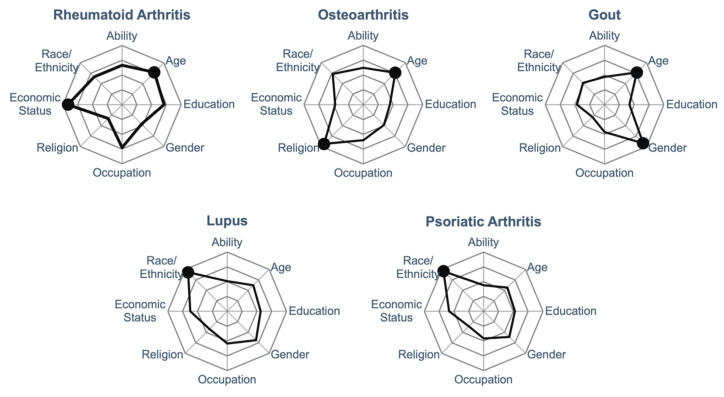
Student identified characteristics associated with each assigned paper. Using a Likert scale from 1 = strongly disagree (center point), to 5 = strongly agree (outer ring), students indicated which characteristic were associated with each paper. *n* = 131–133 for each data point.

The original intent of the assignments was to initiate discussion with students regarding health equity and evidence-based practice to improve their cultural humility and discuss how biases and SDOH impact patient care. Although papers were selected based on specific health disparities, students were asked in the post-assignment survey if they agree or disagree that the following characteristics played a role in this paper. The average of student responses from the Likert scales are demonstrated in Figure 1, with characteristics scoring above 4.0 indicated with a black data point. Given the low response rate in year 1, only the data collected from the second cohort (*n* = 136) are presented. Though none of the papers were specifically selected for age, students identified age as a key characteristic in the papers related to RA, OA, and gout. All other leading characteristics align with the reason why the papers were originally selected. Several other characteristics scored between 3 and 4 on the Likert scale and provide an opportunity to discuss intersectionality and the multiple characteristics that affect care for a single patient. 

**Figure 2:**
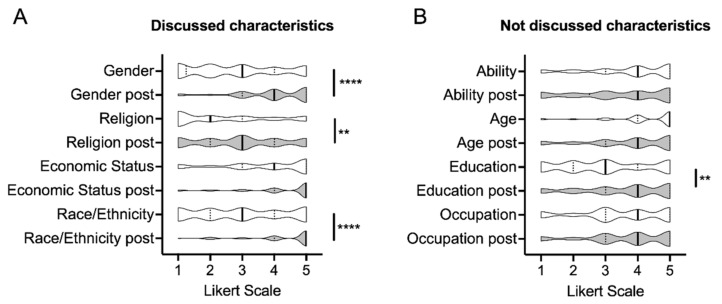
Comparison of student perceptions of how much SDOH affect patient care at baseline and after completing all reading assignments. (**A**) Discussed characteristics in the context of paper assignments, and (**B**) characteristics not discussed in the context of paper assignments. Baseline survey data (*n* = 112) are in white, conclusion survey data (*n* = 101) are in grey. Data are for year 2 cohort only. Mean is denoted by black line within the violin plots with 25% and 75% percentiles denoted in dashed lines. Statistical comparisons were performed by *t*-test. ** (*p* < 0.01), **** (*p* < 0.0001).

After completing each of the assignments and post-assignment surveys, students were asked to complete a final Mentimeter survey using the same questions as the baseline survey. During the first year of conducting this activity, 98 out of 136 (72%) completed the final survey, whereas 101 out of 134 students (75%) completed it during the second year. Data compared between the baseline and final survey indicated that the characteristics discussed in the papers had a significant impact on the students’ perceptions of how certain characteristics affect rheumatology care. Of the characteristics that were discussed (gender, religion, economic status, race/ethnicity) (Figure 2A), students indicated that gender (**** *p* < 0.0001), religion (** *p* < 0.01), and race/ethnicity (**** *p* < 0.0001) exhibited a stronger influence on patient care after completing the assignments as compared to baseline. Economic status also increased but did not reach statistical significance. Of the characteristics that were not discussed in the context of the assigned papers (Figure 2B) (ability, age, education, occupation), only education received a higher score in the concluding survey (** *p* < 0.01). Although students identified age as a factor in three papers, age was not a key component of the post assignment in-class discussion and was not included as a discussed characteristic. 

## 4. Discussion

In this report, we describe the use of health equity literature to initiate discussions about cultural humility and the structural inequities that lead to disproportionate care for marginalized individuals. By first assessing the baseline perception of SDOH, areas of improvement were identified, as not all cohorts in all institutions will have the same perceptions due to cohort demographics, additional cultural humility discussions, and ongoing social movements. Indeed, within our institution, two consecutive cohorts had statistically significant differences in specific perceptions. From these baseline perceptions, a longitudinal approach that includes reading assignments integrated into the curriculum may be developed to initiate discussions of these topics with the goal of improving student perceptions of how specific SDOH affect care. Indeed, we found that students’ perceptions of SDOH improved when engaging with scholarly articles that are complementary to the course content. Such activities are critical to empower students with the tools to continue their own self-education toward cultural humility, which enables them to practice with the most significant impact on patients of all backgrounds. A similar strategy was developed as a standalone activity for medical students to improve cultural humility with LGBTQ patients [21]. Additionally, scientific journal clubs are ubiquitous among graduate programs, but these activities are typically not included as a longitudinal assessment of cultural humility, nor are they often included within a pharmacy course to complement the disease-specific content. 

The Accreditation Council for Pharmacy Education (ACPE) Standards 2016 highlight the importance of cultural awareness and exposure to diverse populations within the pharmacy curriculum, but strategies to implement such activities remain ill-defined [22]. Henson and Drame have discussed the need for intentional, integrative, and comprehensive implementation of health disparities and cultural competency training within the pharmacy curriculum [22]. Within pharmacy curricula, various approaches to achieve these goals have been studied [23] but have generally been included as elective stand-alone courses [24], within skills-based courses [25], or during experiential education [26]. These examples of cultural humility in the curriculum have been shown to be successful, as demonstrated through survey data, indicating improved student awareness. Although the inclusion of cultural humility content in the curriculum in this format is important, the integration of cultural humility content in foundational pharmacology and pharmacotherapy courses provides critical insight into SDOH from the initial exposure to disease-specific content to establish a foundation for consideration as they enter the practicum portion of the curriculum. Additionally, the longitudinal assessment of the effect of all curricular and non-curricular activities has been developed across a four-year pharmacy curriculum [27]. As educators, it is imperative that we provide holistic training for our students to succeed in their future professions, and in the context of pharmacy, this includes equipping students with an understanding of the therapeutics and pharmacology associated with patient care, as well as a foundation to understand SDOH that impact said care. Although it is impossible to educate students about the new therapeutics that will be on the market in 20 years, it is equally unlikely that we will be able to provide comprehensive information to prepare students for every demographic and SDOH that they will encounter throughout their careers. Therefore, the intentional integration of cultural humility content within a disease-specific pharmacy course provides students with a framework, similar to their therapeutics content, for future self-guided education. Notably, the survey questions do not individually connect to cultural humility, but provide a starting point to initiate a dialogue with students about the strengths, weakness, and importance of engaging with the scientific literature focused on SDOH in patient care.

Although the primary goal of this activity was to help shift student perceptions of cultural humility in the context of a pharmacology course, several secondary benefits were observed through the implementation of this activity. Most notably, this strategy prepared students to engage with scientific literature and critically consider the experimental design described in each study. The data indicate that the students are eager to think critically about the intricacies of the research they are consuming and can identify potential biases that arise in the author’s analyses, particularly when the information in the paper is contrary to what they have learned in class. The benefits of such an activity are well documented, particularly in the pharmacy curriculum [28,29,30], as students prepare to implement evidence-based practice with their residency programs and future practice [31,32]. As student pharmacists progress through the curriculum toward APPE rotations and possibly residencies, the lessons learned here will enable the students to read the scientific literature with the goal of not only educating themselves on new therapies, but also how SDOH influence patient care.

Although the goal of this activity was to help shift student perceptions of cultural humility in the context of a pharmacology course, a number of limitations in the methodology and long-term outcomes of this activity remain. First, the baseline and concluding survey data were collected anonymously using Mentimeter, whereas each post-activity survey was collected using a graded assignment. Though the graded assignment was necessary to obtain sufficient survey response rates, the students might have felt obligated to respond according to expectations rather than their own personal perceptions. Second, as with most curricular activities, this activity was designed to educate students in preparation for future practice but did not directly assess that. Instead, this report demonstrates how such an activity can be implemented in a disease-specific pharmacology course and how student perceptions can change using such an activity. Third, the off-target effects of the activity have also not been assessed. Importantly, each manuscript was selected to discuss the role of specific SDOH in patient care relevant to a specific disease within the course, but our data do not provide insight as to whether students’ perceptions impact disease states outside of those intended. If by reading one article and discussing how a specific characteristic affects care, it remains unclear if students’ perceptions of that characteristic affect care in other disease states, both within the course and in other diseases. Finally, it remains unclear if student perceptions of SDOH are affected linearly with their relative enjoyment in reading each of the papers and if the students felt that the activity impacted their knowledge, or attitude of SDOH. Implementing a self-reflection as part of this activity may provide additional insight into the relative impact of using journal articles to improve student perceptions of SDOH in healthcare. Nevertheless, cultural humility and evidence-based practice are critical components of the pharmacy profession and calls for a need of similar activities to help students hone their critical reading skills prior to clinical rotations. Several curricula continue to include such opportunities through scholarship or research courses and electives, however, the implementation of this activity in a disease-specific course serves as an opportunity for instructional scaffolding where students continue to engage with therapeutics content while learning new skills to enrich their educational experience.

## 5. Conclusions

This study describes the intentional integration of a scientific literature activity within a disease-specific course to introduce the social determinants of health and cultural humility training in second year pharmacy students. The semester-long assessment of student perceptions indicates a significant improvement in certain perceptions associated with specific SDOH that were discussed within selected scientific papers. This activity serves as a platform for the instructional scaffolding of disease content, cultural humility, and critical assessment of scientific literature to provide a holistic educational experience for students, which enable continued self-guided learning opportunities for students beyond the pharmacy curriculum.

## Figures and Tables

**Table 1:** Summary of students’ baseline perceptions of how patient characteristics affect rheumatology care in two cohorts of second year pharmacy students.

Characteristic	Year 1 (Mean ± SEM)	Year 2 (Mean ± SEM)	*p* Value
	*n* = 96 (70%)	*n* = 112 (84%)	
Age	3.9 ± 0.12	4.3 ± 0.09	0.15
Ability	3.1 ± 0.14	3.8 ± 0.12	<0.01
Economic Status	3.0 ± 0.13	3.8 ± 0.13	<0.0001
Occupation	3.5 ± 0.13	3.5 ± 0.12	0.95
Education	2.5 ± 0.12	3.0 ± 0.13	0.20
Race/Ethnicity	2.6 ± 0.14	3.0 ± 0.14	0.47
Gender	2.4 ± 0.13	2.8 ± 0.13	0.49
Religion	1.5 ± 0.10	2.3 ± 0.14	<0.001

Data collected using Likert scale: 1 (strongly disagree)—5 (strongly agree). Statistical analysis performed by Kolmogorov-Smirnov t-test.

## Data Availability

The data presented in this study are available on request from the corresponding author.
